# Human Thymic Involution and Aging in Humanized Mice

**DOI:** 10.3389/fimmu.2020.01399

**Published:** 2020-07-07

**Authors:** Qing-Yue Tong, Jue-Chao Zhang, Jing-Long Guo, Yang Li, Li-Yu Yao, Xue Wang, Yong-Guang Yang, Li-Guang Sun

**Affiliations:** ^1^Key Laboratory of Organ Regeneration & Transplantation of the Ministry of Education, The First Hospital of Jilin University, Changchun, China; ^2^National-local Joint Engineering Laboratory of Animal Models for Human Diseases, Changchun, China; ^3^International Center of Future Science, Jilin University, Changchun, China

**Keywords:** thymus involution, aging, human, humanized mouse, recent thymic emigrants

## Abstract

Thymic involution is an important factor leading to the aging of the immune system. Most of what we know regarding thymic aging comes from mouse models, and the nature of the thymic aging process in humans remains largely unexplored due to the lack of a model system that permits longitudinal studies of human thymic involution. In this study, we sought to explore the potential to examine human thymic involution in humanized mice, constructed by transplantation of fetal human thymus and CD34^+^ hematopoietic stem/progenitor cells into immunodeficient mice. In these humanized mice, the human thymic graft first underwent acute recoverable involution caused presumably by transplantation stress, followed by an age-related chronic form of involution. Although both the early recoverable and later age-related thymic involution were associated with a decrease in thymic epithelial cells and recent thymic emigrants, only the latter was associated with an increase in adipose tissue mass in the thymus. Furthermore, human thymic grafts showed a dramatic reduction in *FOXN1* and *AIRE* expression by 10 weeks post-transplantation. This study indicates that human thymus retains its intrinsic mechanisms of aging and susceptibility to stress-induced involution when transplanted into immunodeficient mice, offering a potentially useful *in vivo* model to study human thymic involution and to test therapeutic interventions.

## Highlights

– Human thymus/HSPC-grafted hu-mice show robust thymopoiesis and T cell development.
– Human thymus is susceptible to stress-induced involution in hu-mice.
– Human thymus retains its intrinsic mechanisms of aging in hu-mice.

## Introduction

Aging is a continuous process that is associated with increased susceptibility to infection, autoimmunity, and cancer (1, 2). The thymus gland is an essential lymphoid organ responsible for the production of T cells (3, 4). Thymic involution, or the shrinking of the thymus with age, is common in all species possessing a thymus. However, most of what we know about thymic aging is based on mouse studies, which is unlikely to be identical to humans. It has been shown that naïve T cells from young and aged mice comparable amounts of T-cell receptor excision circles (TREC), whereas the TREC content of naïve human T cells is high in neonates and declines with age (5). Another difference is that thymic output maintains naïve T cell populations in mice, whereas human T cells may divide in the periphery without losing their naïve phenotype as currently defined (6). Thus, mouse studies may offer limited insights into the process and underlying mechanisms of human thymic aging.

Previous snapshot studies of human thymic tissues suggest that the human thymus grows from birth to 2–3 years of age, followed by involution throughout the period of adolescence (4), and a weight decrease of several fold by the age of 50–60 years (7). Although these snapshot studies provide some insight into the aging of the human thymus, the nature of this process in humans is still largely unexplored due to the lack of a model system that permits longitudinal studies of human thymic involution. Transplantation of human fetal thymic tissue (under renal capsule) and fetal liver-derived CD34^+^ cells (i.v.) achieves efficient human thymopoiesis and T cell development in immunodeficient mice (8–10). The reconstituted mice showed sustained repopulation with multilineages of human lymphohematopoietic cells, including T, B and dendritic cells, and the formation of secondary lymphoid organs. The engrafted human thymus was found to consist of human thymocytes with a normal phenotypic distribution of double negative, double positive, CD4 single positive, and CD8 single positive cell populations (9). Here, we sought to understand the involution and aging of human thymus in this humanized mouse (hu-mice) model. We found that human thymus in hu-mice undergoes both stress-induced and age-related thymic involution, suggesting that the hu-mouse model may be useful for understanding human thymic involution and testing therapeutic interventions.

## Materials and Methods

### Mice and Human Samples

NOD.CB17-*Prkdc*^scid^/ NcrCrl (NOD/SCID) mice were purchased from Beijing Vital River Laboratory Animal Technology Co. Ltd., and were housed in a specific pathogen-free micro-isolator environment and used in experiments at ~5 weeks of age. Discarded human fetal tissues with gestational age of 17 to 20 weeks and human blood samples were obtained with informed consent at the First Hospital of Jilin University. Protocols involved in the use of human tissues and animals were reviewed and approved by the Institutional Review Board and Institutional Animal Care and Use Committee of the First Hospital of Jilin University, and all of the experiments were performed in accordance with the protocols.

### Construction of Humanized Mice

Hu-mice were constructed by transplantation of human fetal tissues (~1 mm^3^ in size, under renal capsule) and fetal liver-derived CD34^+^ cells (i.v.; 3 × 10^5^/mouse) from the same fetal donor into 2 Gy-irradiated NOD/SCID mice as previously described (9, 11).

### Flow Cytometric Analysis

Human immune cell reconstitution in hu-mice was analyzed by flow cytometry (FCM) using various combinations of the following monoclonal antibodies: anti-human CD45, CD3, CD4, CD8, CD45RA, CD45RO, CD69, CCR7, CD31 (all purchased from Biolegend, San Diego, CA, USA); and anti-mouse CD45 (BD Pharmingen) and Ter119 (Biolegend, San Diego, CA, USA). Peripheral blood was collected from tail vein into heparinized tubes, and mononuclear cells were purified by density gradient centrifugation with Histopaque-1077 (Sigma-Aldrich, St. Louis, MO, USA). FCM analysis was performed on a FACS Fortessa (BD Biosciences). Dead cells were excluded from the analysis by gating out lower forward scatter and high propidium iodide–retaining cells. Data analysis was performed using FlowJo 10.3 software.

### Immunofluorescence and Histological Analysis

Human thymic grafts were embedded in tissue freezing medium (OCT compound-embedding medium for frozen specimens; Miles Laboratories, Elkart, IN, USA) and immediately frozen in liquid nitrogen and then stored at −80°C. Cryosections (3.5 μm) were prepared and fixed in cold acetone for 20 min. The sections were stained with Alexa Fluor 647 anti-human CD326 (EpCAM) antibody (clone 9C4, 1:200; Biolegend, San Diego, CA, USA), followed by DAPI staining. The slides were imaged and processed using a fluorescence microscope. To evaluate fat deposition, thymic sections were stained with Oil Red O (for visualizing fat deposition) and hematoxylin. Images of the sections were collected using a light microscope (Olympus Corporation) from four different fields at ×100 magnification. Image Pro Plus software was used to analyze the integrated optical density (IOD) of the Oil Red O-stained areas.

### Real-Time PCR

Total RNA was extracted with Trizol (Invitrogen, Waltham, MA, USA), and cDNA was synthesized using TransScript First-Strand cDNA Synthesis SuperMix (TransGen Biotech, Beijing, China). Quantitative real-time PCR was performed using a SYBR Green Kit (TransGen Biotech) with a StepOnePlus Real-Time PCR System (Applied Biosystems, Inc., Carlsbad, CA, USA). Quantitative real-time PCR primer sequences used in this study are shown in Table 1 (12, 13). Reactions were performed in triplicate in three separate experiments. Relative gene expression was normalized to EpCAM.

**Table 1:** Quantitative real-time PCR primer sequences.

**Genes**	**Forward (5^**′**^-3^**′**^)**	**Reverse (5^**′**^-3^**′**^)**
*FOXN1*	TCCCTCACTCACTGACTTCG	GTGGCATCGAAGATGATGTC
*AIRE*	GATGACCTGGAGTCCCTTCT	CTCATCAGAGCTGCATGTCC
*EpCAM*	AATCGTCAATGCCAGTGTACTT	TCTCATCGCAGTCAGGATCATAA
SJ TREC	CAC ATC CCT TTC AAC CAT GCT	TGC AGG TGC CTA TGC ATC A
TRAC	TGG CCT AAC CCT GAT CCT CTT	GGA TTT AGA GTC TCT CAG CTG GTA CAC

### Real-Time PCR for Relative TREC Expression

DNA was purified from human PBMCs and humanized mouse PBMCs and spleen cells using the QIAamp DNA Blood Mini Kit according the manufacturer's instructions (Qiagen, Hilden, Germany). Quantitative real-time PCR was performed using AceQ Universal U+ Probe Master Mix V2 (Vazyme Biotech) with a StepOnePlus Real-Time PCR System (Applied Biosystems, Inc., Carlsbad, CA, USA). Quantitative real-time PCR primer and probe sequences used in this study are shown in Tables 1, 2. Reactions were performed in triplicate in three separate experiments. Relative gene expression was normalized to TRAC. (14)

**Table 2:** Quantitative real-time PCR probe sequences.

**Genes**	**Sequences (5^**′**^-3^**′**^)**
SJ TREC probe	FAM-ACA CCT CTG GTT TTT GTA AAG GTG CCC ACT TAMRA
TRAC probe	FAM-TCC CAC AGA TAT CCA GAA CCC TGA CCCTAMRA

### Statistical Analysis

All data are presented as mean ± S.D and statistical significances between two groups were calculated using unpaired, non-parametric, two-tailed Student's *t*-test. Differences with a *P*-values of <0.05 were considered statistically significant. Statistical analysis was performed using GraphPad Prism 5 software and Microsoft Office Excel 2013.

## Results

### Age-Associated Decrease in CD4^+^ naïve T Cells and Recent Thymic Emigrants in Healthy Humans

**Figure 1:**
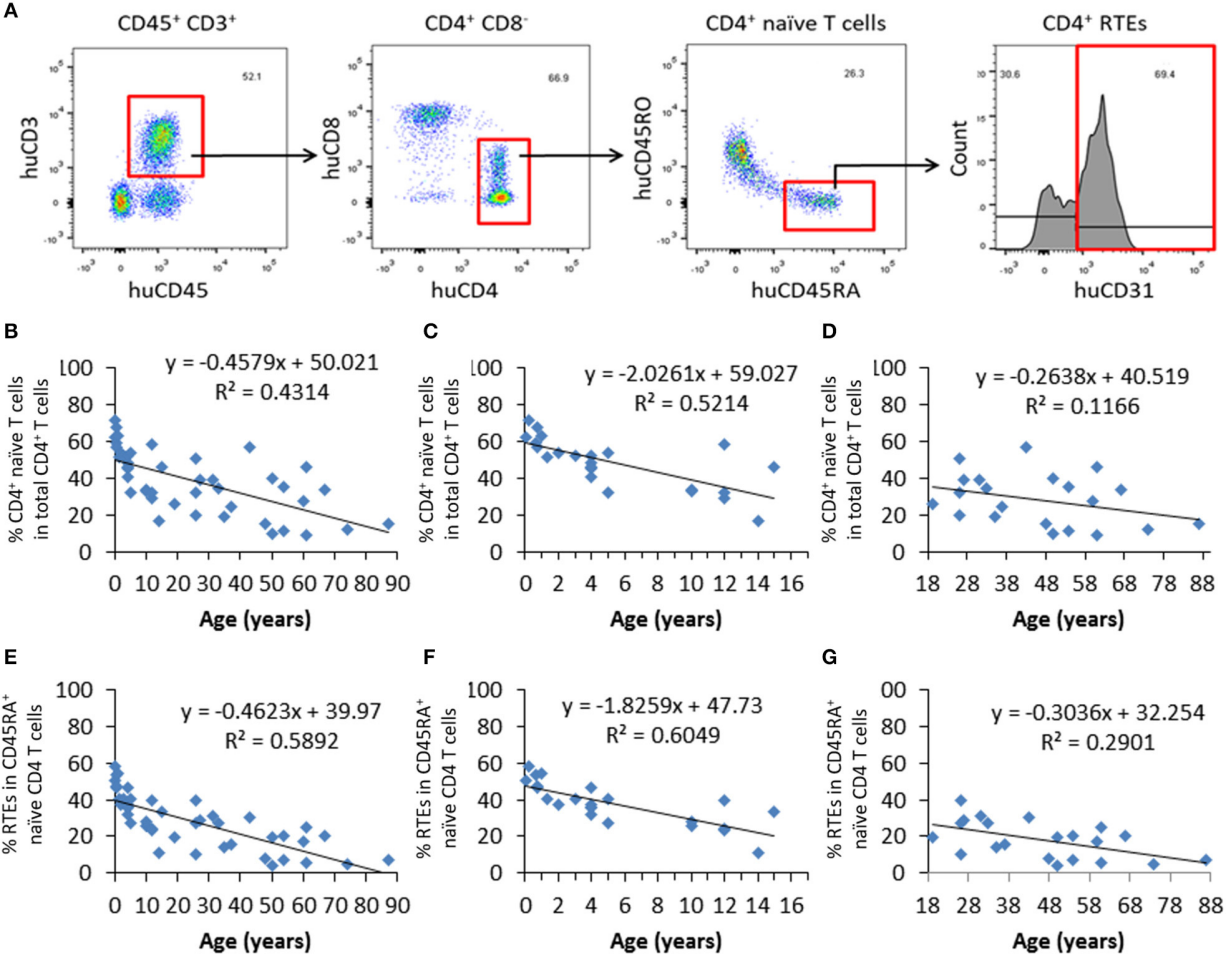
Progressive decrease in CD4^+^ naïve T cells and RTEs with age in healthy humans. PBMCs from 44 healthy individuals were analyzed for the ratios of CD4^+^ naïve T cells and RTEs. **(A)** FCM profiles showing the gating strategy for identifying CD4^+^ naïve (CD4^+^CD45RA^+^CD45RO^−^) T cells and RTEs (CD4^+^CD45RA^+^CD45RO^−^CD31^+^). **(B–D)** Percentages of naïve CD4^+^ T cells in all individuals analyzed (n=44; B), individuals from newborn to 17 years old (*n* = 23; **C**), and individuals from 18 to 87 years old (*n* = 21; **D**). **(E–G)** Percentages of CD4^+^ RTEs in all individuals analyzed (*n* = 44; **E**), individuals from newborn to 17 years old (*n* = 23; **F**), and individuals from 18 to 87 years old (*n* = 21; **G**).

Blood samples from 44 healthy individuals (age 31 days to 87 years) were analyzed for the ratios of CD4^+^ naïve T cells and recent thymic emigrants (RTEs) by flow cytometry (FCM), in which CD4^+^ naïve T cells and RTEs were identified as CD4^+^CD45RA^+^CD45RO^−^ and CD4^+^CD45RA^+^CD45RO^−^CD31^+^, respectively (Figure 1A). We found that there was an age-associated decline in CD4^+^ naïve T cells (Figure 1B), while such tendency was not detected in the levels of total CD4^+^ T cells (Figure S1). Consistent with previous studies (15), the slope of decline was sharper for the individuals up to the end of puberty (from 1 month to 15 years old) than those 19–87 years old (Figures 1C,D). Similar to CD4^+^ naïve T cells, CD4^+^ RTEs also showed a progressive age-associated decrease (Figure 1E), with a much sharper decline for individuals before the end of puberty (Figures 1F,G). For the levels of both naïve T cells and RTEs, the coefficient of correlation with age was significantly higher for individuals aged from 1 month to 15 years old than those 19–87 years old (*p* < 0.0001 and *p* < 0.0001 for native T cells and RTEs, respectively). These data are in agreement with previous reports (16, 17) confirming that CD4^+^ naïve T cells and CD4^+^ RTEs are adequate measures of thymic output potential (Figure 1).

### Kinetic Changes in Human CD4^+^ naïve T Cells and RTEs in Humanized Mice

**Figure 2:**
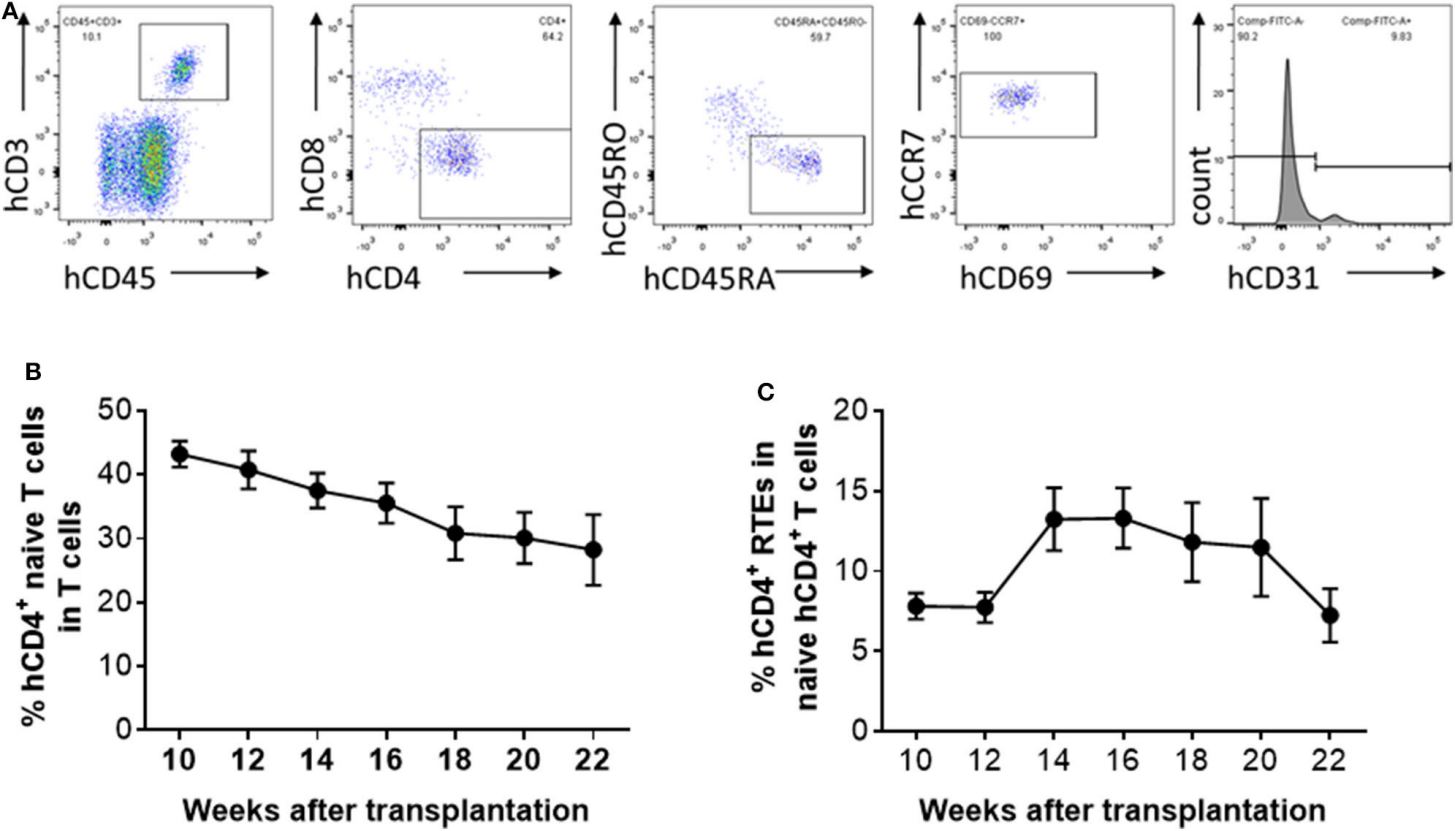
Kinetic changes in human CD4^+^ naïve T cells and RTEs in hu-mice. **(A)** Representative FCM profiles. **(B,C)** Percentages of CD4^+^CD45RA^+^CD45RO^−^ naïve T cells **(B)** and CD4^+^CD45RA^+^CD45RO^−^CD31^+^ RTEs **(C)** in PBMCs at the indicated times (*n* = 5–28 animals were analyzed at each time point).

Peripheral blood was collected from hu-mice at 10, 12, 14, 16, 18, 20, and 22 weeks after human thymus and CD34^+^ cell transplantation, and analyzed for human CD4^+^ naïve T cells and CD4^+^ RTEs (Figure 2A). The percentage of CD4^+^ naïve T cells showed a relatively steady decline during the observation period of 22 weeks (Figure 2B). However, the kinetics of the naïve T cell levels were not coincident with the kinetics of the RTE levels. The percentage of CD4^+^ RTEs in T cells was low until 12 weeks, and then increased by nearly 2-fold between 12 and 14 weeks (Figure 2C). CD4^+^ RTE levels remained similar between 14 and 16 weeks, and declined progressively thereafter (Figure 2C). The low percentage of CD4^+^ RTEs early after humanization may reflect the recovery process of the transplanted thymic tissue. However, RTE levels in naïve CD4^+^ T cells in hu-mice were surprisingly low in general compared to those of humans (Figure 1). It has been reported that human T cells can divide in the periphery without losing their naïve phenotype (6). Thus, although the lower RTE levels in hu-mice may be due to inefficient T cell output of the human thymic graft, it may also be attributed to increased homeostatic proliferation of naïve human T cells in hu-mice.

### Aging of Human Thymic Grafts in Humanized Mice

**Figure 3:**
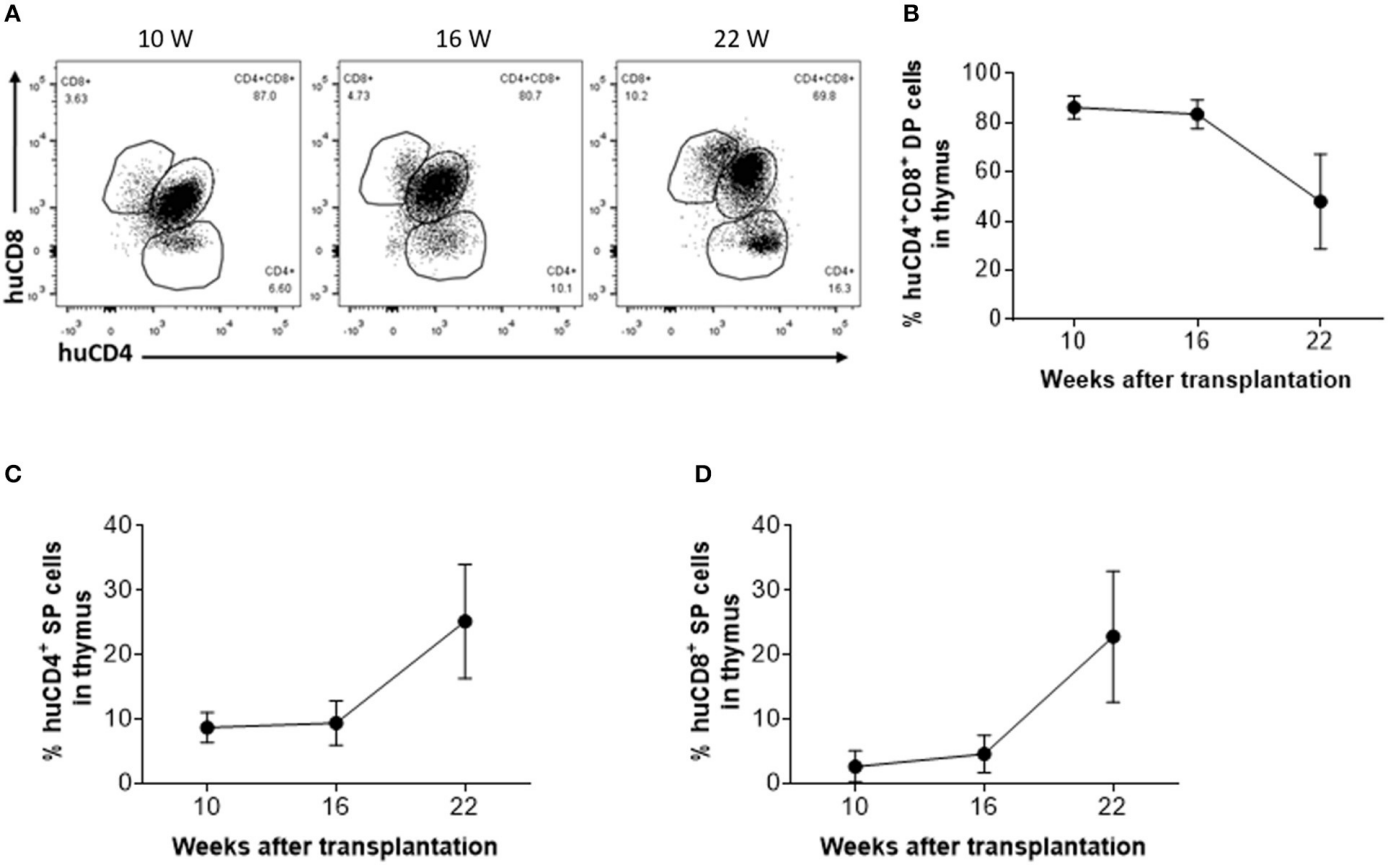
Phenotypic distribution of human thymocytes in humanized mice. **(A)** Representative FCM profiles of CD4 and CD8 expression on human thymocytes. **(B–D)** Percentages of DP **(B)**, CD4 SP **(C)** and CD8 SP **(D)** thymocytes at the indicated time points (*n* = 3–4 animals were analyzed at each time point).

To determine whether thymic aging is responsible for the observed decline in CD4^+^ RTE levels 16 weeks after thymic transplantation (Figure 2C), human thymic grafts were harvested 10, 16, and 22 weeks after transplantation and analyzed for thymopoiesis and involution (Figure 3A). Although a significant population of CD4^+^CD8^+^ double-positive (DP) thymocytes was detected in the human thymic grafts, a significant decline in the ratio of DP thymocytes, which was associated with an increase in CD4 single positive (SP) and CD8 SP thymocytes, was clearly detected between 16 and 22 weeks (Figures 3B–D).

**Figure 4:**
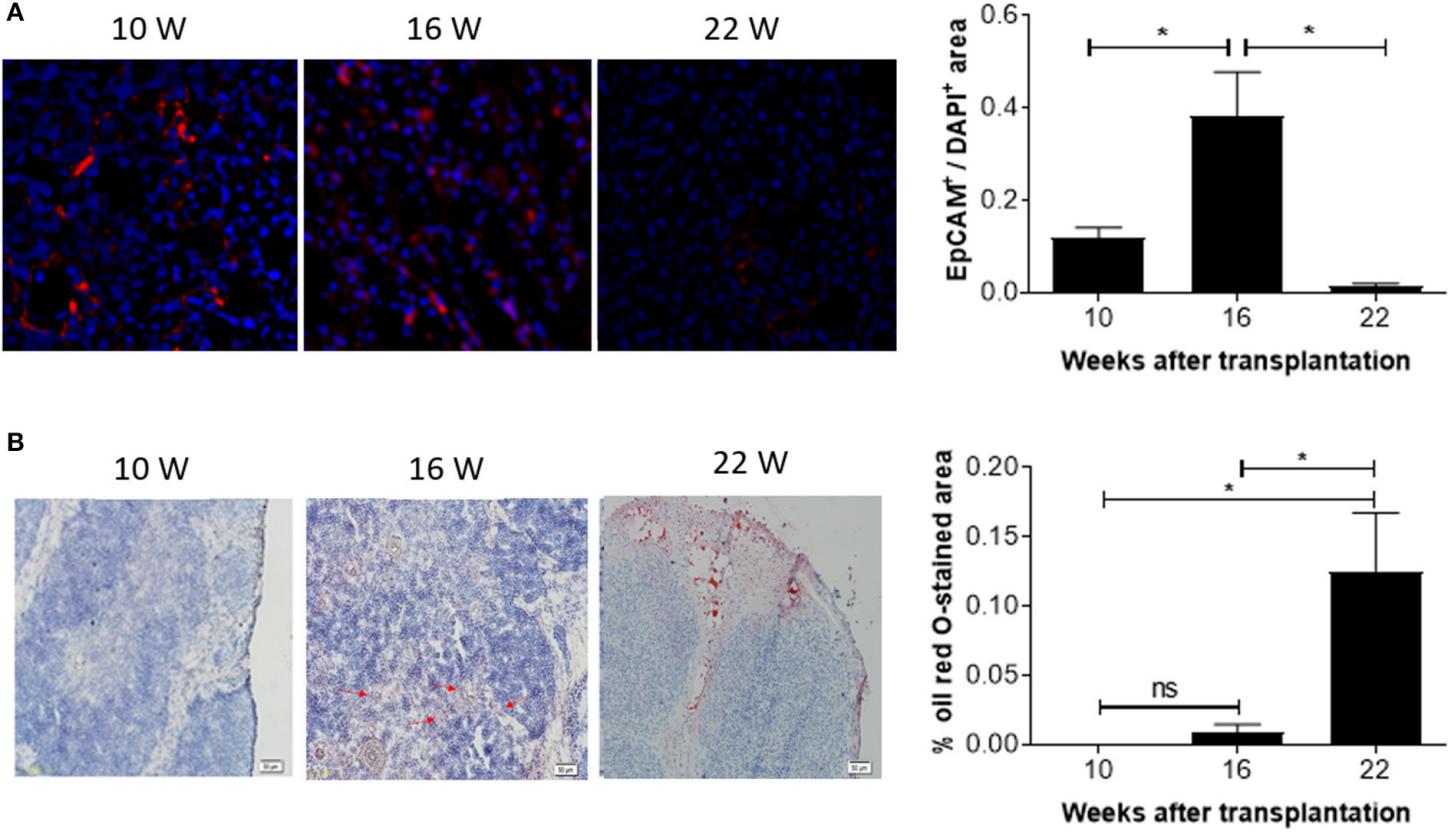
Kinetic changes in EpCAM^+^ TECs and fat-deposition in human thymic grafts from hu-mice. Tissue sections of human thymic grafts prepared from hu-mice at weeks 10, 16, and 22 were stained with anti-EpCAM antibody and DAPI (**A**; *n* = 3–6 at each time point) or with Oil Red O and Hematoxylin (**B**; *n* = 4–5 animals at each time point). **(A)** Images of representative samples (left) and ratios of EpCAM^+^ to DAPI^+^ areas (right). **(B)** Images of representative samples (left) and percentages of Oil Red O-stained areas (right). Data are presented as mean ± SEM. *p < 0.05.

The observed decline in DP thymocytes suggests an age-associated decrease in the function of the human thymic grafts in hu-mice. To confirm this, we next measured the number of EpCAM-positive thymic epithelial cells (TECs) in the human thymic grafts. Immunofluorescence staining revealed that EpCAM-positive TECs increased by ~3-fold between 10 and 16 weeks, and then declined progressively (by over 20-fold between 16 and 22 weeks; Figure 4A). In line with this observation, RT-qPCR analysis revealed a similar increase in EpCAM expression between weeks 10 and 16 and a substantial decline between weeks 16 and 22 (Figure S2). The early increase in TECs presumably reflects the recovery process of the human thymic graft. However, the later decrease in TECs is likely to be the consequence of thymic involution.

We also measured changes in adipose tissue mass, another indicator of age-related thymic involution (18). Oil Red O staining revealed a progressive increase in lipid-laden cells. Lipid-laden cells were not detected or barely detectable in the human thymic grafts at weeks 10 and 16, but a dramatic increase (over 10-fold) was detected at week 22 (Figure 4B). Together, our data suggest that significant age-related involution of human thymus occurred 16 weeks after transplantation.

**Figure 5:**
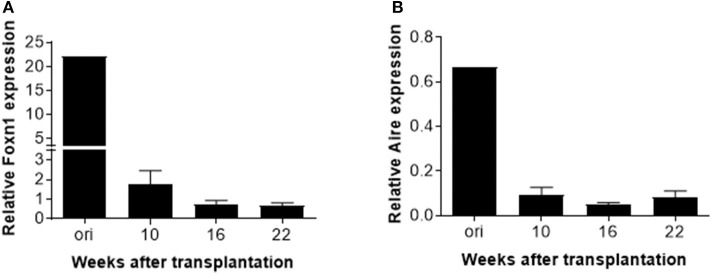
*AIRE* and *FOXN1* expression in TECs in human thymic grafts. Human thymic grafts prepared from hu-mice at weeks 10, 16 and 22 were analyzed for *FOXN1, AIRE*, and EpCAM gene expression by real-time RT-PCR analysis. Relative expression levels (normalized to EpCAM) of *FOXN1* **(A)** and *AIRE* **(B)** genes shown as the mean ± SEM (*n* = 3–4 animals were analyzed at each time point).

Finally, real-time PCR was performed to measure expression of transcriptional factor forkhead box protein N1 (Foxn1) and autoimmune regulator (Aire) in the human thymic grafts. We found that *FOXN1* expression in the human thymic grafts harvested at week 10 was considerably lower than in the original human fetal thymus (prior to transplantation into mice; Figure 5A and Figure S3A). These results suggest that rapid aging or involution occurred following thymic transplantation into mice, and/or that the mouse xenogeneic environment may not provide optimal conditions to support human thymic epithelial cell (TEC) function. A further decrease in *FOXN1* expression was seen between 10 and 16 weeks (Figure 5A), likely reflecting an age-related thymic involution. Like *FOXN1*, there was a marked loss of AIRE expression in human thymic grafts by 10 weeks after transplantation, and *AIRE* expression remained low throughout the observation period of 22 weeks (Figure 5B and Figure S3B).

### Relative TREC Expression Between Human and Humanized Mice

**Figure 6:**
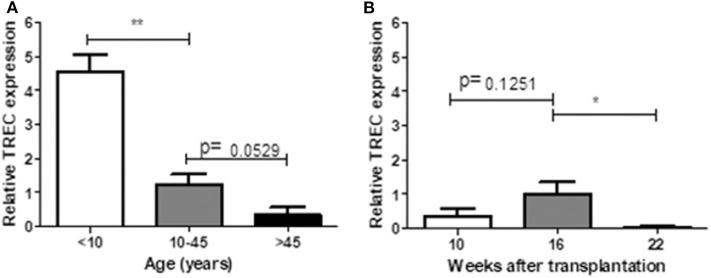
Relative TREC expression between human and humanized mice. PBMCs prepared from 14 healthy individuals **(A)** and hu-mice at 10^th^, 16^th^, and 22^nd^ week **(B)** were analyzed for the relative TREC expression by real-time PCR. Relative expression levels (normalized to TRAC) shown as the mean ± SEM (*n* = 3–7 human samples were analyzed at each age range, *n* = 3–4 animals were analyzed at each time point). Data are presented as mean ± SEM. *p < 0.05 and **p < 0.01.

TRECs are stable episomal, non-replicative DNA circles generated during T-cell receptor gene rearrangement in developing T-lymphocytes in the thymus. Therefore, TRECs are a marker for recently formed T-lymphocytes (19). We measured relative expression of TRECs in human (in different age ranges) and hu-mice (10, 16, and 22 weeks after transplantation). In line with previous reports (20, 21), human PBMCs showed a clear age-dependent decline in TRECs (Figure 6A). In hu-mice, relative TREC expression showed a moderate increase from week 10 to week 16, followed by a significant decline at week 22 week (Figure 6B), suggesting an age-associated decrease of thymic output after 16 weeks.

## Discussion

The thymus is a primary lymphoid organ where T cells are generated. Thymic involution, or the shrinking of the thymus with age, is an important factor that inhibits thymic output, leading to immune defects in vertebrates. Although thymic atrophy or involution has been extensively investigated in animal models, in humans the process and its underlying mechanisms remain relatively unknown, largely due to the lack of an *in vivo* model for longitudinal studies. The lack of a suitable *in vivo* model is also a bottleneck in developing interventions to treat thymic involution. In this study, we explored the potential to study human thymic involution in hu-mice constructed by transplantation of human thymic tissue and hematopoietic stem/progenitor cells into immunodeficient mice.

Thymic involution with age is a chronic process that results in progressive reduction in thymic output, leading to degeneration of adaptive immunity in aged individuals. In addition to age-related involution, the thymus may undergo acute (recoverable) atrophy under certain stress conditions, such as infection, pregnancy, and chemotherapy. In hu-mice, we found that human thymus first undergoes acute recoverable involution, followed by an age-related chronic form of involution. The first thymic involution was likely caused by transplantation stress, and recovered by week 14. Although the early recoverable atrophy (likely induced by transplantation stress) and the later age-related thymic involution were both associated with a decrease in TECs and RTEs, only the latter was associated with an increase in adipose tissue mass in the thymus. This is consistent with previous reports in mice that age-related thymic involution is closely associated with adipocyte expansion in the thymus (22).

FOXN1 appears in the sixth week of gestation in humans, serving as a key regulator of TECs development and differentiation in the fetal and adult thymus (23). Snapshot analysis of thymic tissues revealed that thymic *FOXN1* transcription correlates with age, with a sharp decline after adolescence (23). Mouse studies revealed that the frequency of FOXN1^+^ TECs declines rapidly within a few weeks after birth and remains constant up to 2 years of age, while FOXN1 downregulation continues on a per-cell basis through the end of life (24). AIRE is another key transcription factor; it is expressed in mTECs at the final maturation stage and also shows downregulation with age (25, 26). We found that the expression levels of both *FOXN1* and *AIRE* decreased substantially by 10 weeks after transplantation, when compared to the original fetal thymic tissue. Since FOXN1^+^ and AIRE^+^ TECs are highly sensitive to toxic or damaging insults (24, 27), both transplantation stress and aging factors may contribute to the observed early drop in FOXN1 and AIRE expression in the human thymic grafts. We acknowledge that this study did not investigate whether changes in these transcription factors are different among different types of TECs.

We noted that thymic output, as determined by measuring RTEs, is considerably lower in hu-mice compared to humans. It has been reported that human T cells can divide in the periphery without losing their naïve phenotype (6). Thus, although the lower RTE levels in hu-mice may be due to inefficient T cell output of the human thymic graft in the mouse environment, it may also be attributed to an increase in homeostatic proliferation of naïve human T cells in hu-mice. Regardless, further analysis of the thymic grafts indicated that the frequency of RTEs is correlated with thymic function, and thus can be used as an indicator of thymic involution. TREC is another measure of thymic function. It can be used to estimate thymic activity in peripheral blood because intrathymic and peripheral TREC values are correlated (20). We measure relative TREC expression of hu-mice at different time points after transplantation. Relative TREC expression was correlated the ratio of RTEs, with a moderate increase from week 10 to week 16, followed by a significant decrease at week 22. These results suggest that the human thymus went through a recovery process until 16 weeks after transplanted into the mice and began to involute thereafter.

In conclusion, this study suggests that human thymus in hu-mice undergoes both stress-induced acute thymic involution and age-related chronic thymic involution. We acknowledge that the hu-mouse host environment is not identical to that of a human. However, previous studies from our group and others have confirmed that human thymic grafts remain functional in this hu-mouse model (8, 9, 28). Furthermore, the current study provides a proof-of-principle, that human thymic grafts retain their intrinsic mechanisms of thymic aging and susceptibility to stress-induced acute involution. Thus, this hu-mouse model offers a potentially useful *in vivo* system for understanding human thymic involution and testing therapeutic interventions.

## Data Availability Statement

All datasets generated for this study are included in the article/Supplementary Material.

## Ethics Statement

The studies involving human participants were reviewed and approved by Medical Ethics Committee of the First Hospital of Jilin University. Written informed consent to participate in this study was provided by the participants' legal guardian/next of kin. The animal study was reviewed and approved by Institutional Animal Care and Use Committee of the First Hospital of Jilin University.

## Author Contributions

Q-YT, J-CZ, J-LG, and YL performed experiments and analyzed data. L-YY, XW, and Y-GY discussed the data and critically reviewed the manuscript. Q-YT and L-GS conceptualized the project and wrote the manuscript. All authors contributed to the article and approved the submitted version.

## Conflict of Interest

The authors declare that the research was conducted in the absence of any commercial or financial relationships that could be construed as a potential conflict of interest.

## Supplementary Material

Figure S1 CD4+ T cells are unchanged with age in healthy humans. PBMCs from 44 healthy individuals were analyzed for the ratios of CD4+ T cells.

Figure S2 EpCAM expression in human thymic grafts. Human thymic grafts prepared from hu-mice at weeks 10, 16, and 22 were analyzed for EpCAM gene expression by real-time RT-PCR analysis. Relative expression levels (normalized to β-actin) of EpCAM gene shown as the mean ± SEM (*n* = 3–4 animals were analyzed at each time point).

Figure S3 AIRE and FOXN1 expression in human thymic grafts. Human thymic grafts prepared from hu-mice at weeks 10, 16, and 22 were analyzed for AIRE and FOXN1 gene expression by real-time RT-PCR analysis. Relative expression levels (normalized to β-actin) of FOXN1 **(A)** and AIRE **(B)** genes shown as the mean ± SEM (*n* = 3–4 animals were analyzed at each time point).
