# Supplementary figures and images for: Human Thymic Involution and Aging in Humanized Mice
Source: Front Immunol. 2020 Jul 7;11:1399. doi: 10.3389/fimmu.2020.01399 (PMC7358581; doi:10.3389/fimmu.2020.01399)

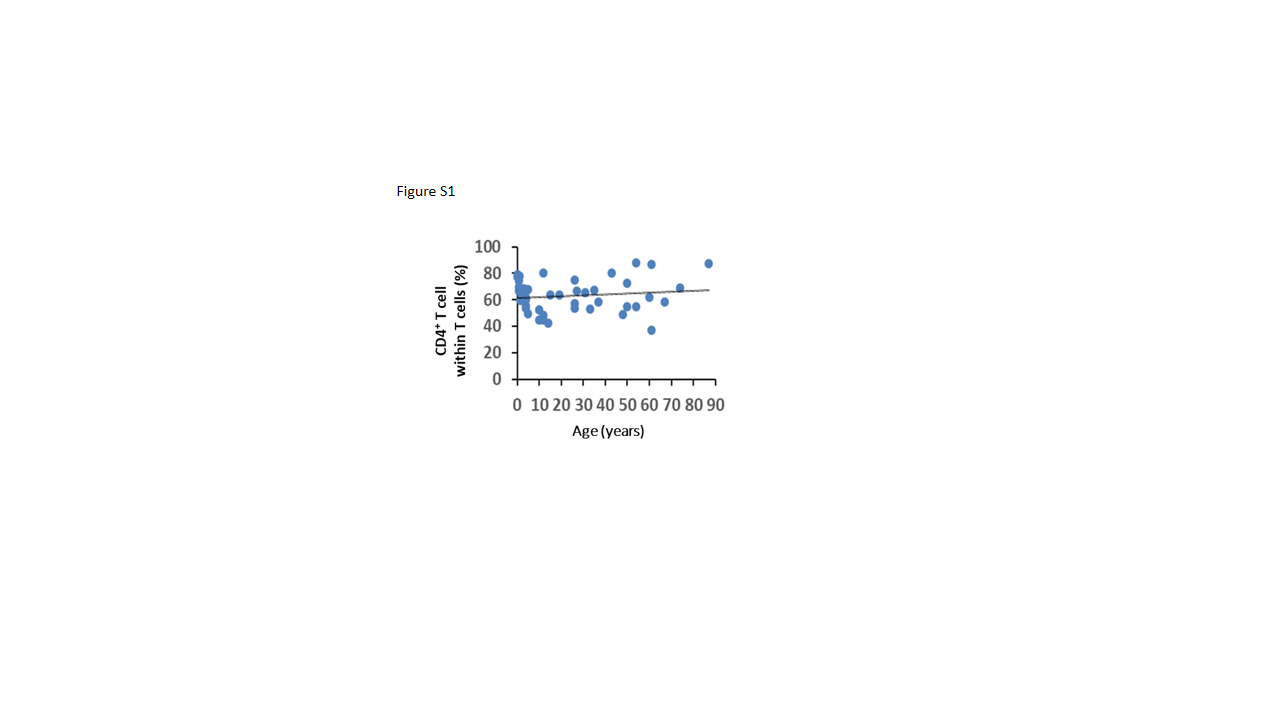

Supplement: Figure S1 — CD4+ T cells are unchanged with age in healthy humans. PBMCs from 44 healthy individuals were analyzed for the ratios of CD4+ T cells. [file Image_1.tif]

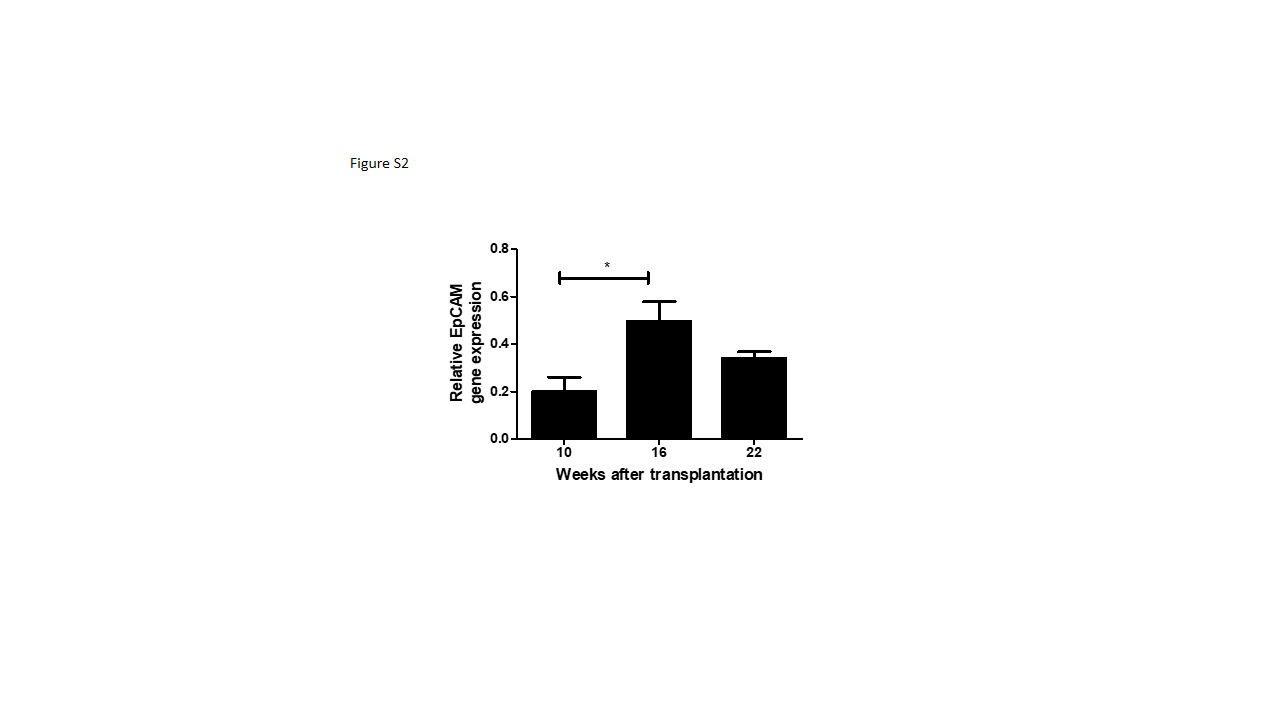

Supplement: Figure S2 — EpCAM expression in human thymic grafts. Human thymic grafts prepared from hu-mice at weeks 10, 16, and 22 were analyzed for EpCAM gene expression by real-time RT-PCR analysis. Relative expression levels (normalized to β-actin) of EpCAM gene shown as the mean ± SEM (n = 3–4 animals were analyzed at each time point). [file Image_2.tif]

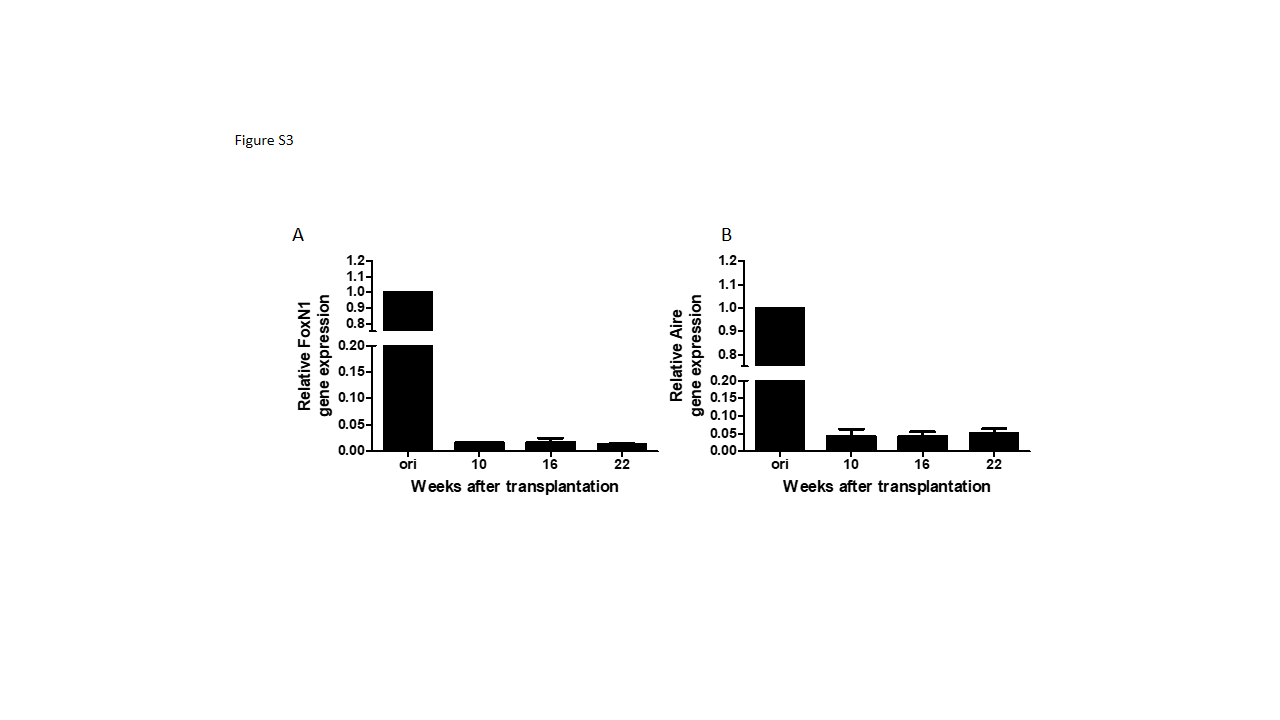

Supplement: Figure S3 — AIRE and FOXN1 expression in human thymic grafts. Human thymic grafts prepared from hu-mice at weeks 10, 16, and 22 were analyzed for AIRE and FOXN1 gene expression by real-time RT-PCR analysis. Relative expression levels (normalized to β-actin) of FOXN1 (A) and AIRE (B) genes shown as the mean ± SEM (n = 3–4 animals were analyzed at each time point). [file Image_3.tif]
